# Supplementary material for: Field-programmable silicon temporal cloak
Source: Nat Commun. 2019 Jun 20;10:2726. doi: 10.1038/s41467-019-10521-5 (PMC6586806; doi:10.1038/s41467-019-10521-5)
Supplement: Supplementary file 1 — Supplementary Information [file 41467_2019_10521_MOESM1_ESM.pdf]

Supplementary Material

Field-programmable silicon temporal cloak

Feng Zhou [1], Siqi Yan [1], Hailong Zhou [1], Xu Wang [1], Huaqing Qiu [3], Jianji Dong[1,], Linjie Zhou[2], Yunhong Ding[3], Cheng-Wei Qiu[4,5,*], and Xinliang Zhang[1,*]*

[1]Wuhan National Laboratory for Optoelectronics, School of Optical and Electronic Information, Huazhong University of Science and Technology, 430074, Wuhan, China.

[2]State Key Laboratory of Advanced Optical Communication Systems and Networks, Depart of Electronic Engineering, Shanghai Jiao Tong University, Shanghai 200240, China.

[3]Department of Photonics Engineering, Technical University of Denmark, DK-2800 Kongens Lyngby, Denmark

[4]Department of Electrical and Computer Engineering, National University of Singapore, Engineering Drive 3, Singapore 117583, Singapore.

[5]NUS Suzhou Research Institute (NUSRI), Suzhou Industrial Park, Suzhou 215123, China

*Corresponding Email: jjdong@hust.edu.cn, chengwei.qiu@nus.edu.sg & xlzhang@hust.edu.cn

Supplementary Note 1: Scheme and whole micrograph of ET-MRR

To implement the cloaking system, electrically tuned microring resonator (ET-MRR) is employed as a tunable filter. The ET-MRR consists of a ring waveguide, two straight waveguides, two contacts and three grating couplers. The global micrograph is shown in Supplementary Fig. 1. In the cloaking system, the probe beam is vertically coupled into the ET-MRR from the grating coupler on the left and will output from the drop port.

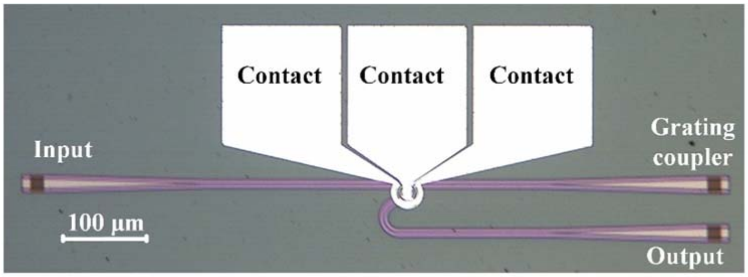

Supplementary Figure 1 | The whole micrograph of ET-MRR.

Supplementary Note 2: Principle of the electrically controllable time lens

The electrically controllable time lens consists of the optical frequency comb generator and the ET-MRR driven by an electrical split sawtooth signal. Ideally, the output optical field $E_{\mathrm{MRR\_S}}(t)$ induced by the electrical sawtooth waveform can be expressed as

$$E_{\mathrm{MRR\_S}}(t)=\exp\left[j(\omega_0+at)t\right]=\exp(j\omega_0 t)\times\exp\left(jat^2\right) \quad (1)$$

where j is the imaginary unit, ω_0 is the angular frequency of the probe light and a is the slope of the sawtooth waveform driving voltage. Supplementary Equation 1 shows that a linear frequency chirp or a quadratic phase modulation is introduced into the probe light. In other words, the optical frequency comb generator together with a sawtooth-waveform-driven ET-MRR can be regarded as a swept-frequency time lens. In order to create a swept-frequency time lens that adapts to the temporal cloak, we change the sawtooth signal to

a split sawtooth signal with a slope, center and duration of a, t_0 and Δt, respectively. Then, the Supplementary Equation 1 is rewritten as

$$E_{\mathrm{MRR\_S}}(t)=\begin{cases}\exp(j\omega_0 t)\times\exp(jat^2)\times\exp(-j(t_0-\Delta t/2)t) & t_0-\Delta t/2<t<t_0\\ \exp(j\omega_0 t)\times\exp(jat^2)\times\exp(-j(t_0+\Delta t/2)t) & t_0<t<t_0+\Delta t/2\end{cases} \quad (2)$$

Supplementary Equation 2 shows that a superior swept-frequency time lens can be created by introducing a driving split sawtooth signal. Obviously, the swept-frequency time lens can be easily opened and closed by setting the split sawtooth and direct current (DC) signal applied on the ET-MRR, respectively. Without loss of generality, the split sawtooth signal is recorded as 1, representing *state on* of swept-frequency time lens, and the DC signal is recorded as 0, representing *state off* of swept-frequency time lens. Then, the electrically controllable time lens is created.

Supplementary Note 3: Simulation of the temporal cloak system

In order to simple verify the feasibility of our cloaking system, we simulate the cloak evolution along different nodes of the periodical temporal cloak system, including temporal waveform at event plane and the output waveforms at the four kinds of states of the temporal cloak.

Firstly, the temporal waveform at the event plane is simulated by analytical method. According to the Supplementary Equation 1, we can see that a split quadratic phase modulation is introduced with a split sawtooth-waveform-driven ET-MRR. However, the output optical field of the ET-MRR, $E_{\mathrm{MRR\_DC}}(t)$, is not monochromatic light in practice when the ET-MRR is driven by a DC voltage, because the ET-MRR has a large bandwidth to contain multiple residual sidebands. Thus, the Supplementary Equation 2 can be rewritten as

$$E_{\mathrm{MRR\_S}}(t)=E_{\mathrm{MRR\_DC}}(t)\times\exp\left(jat^{2}\right) \tag{3}$$

At event plane, the optical field $E_{e}(t)$ can be expressed by

$$E_{\mathrm{e}}(t)=E_{\mathrm{MRR\_S}}(t)*h_{\mathrm{ND}}(\;) \tag{4}$$

where $*$ denotes the convolution operation and $h_{\mathrm{ND}}(t)=\exp\left(jbt^{2}\right)$ is the impulse response of the normal dispersion element, where $b=-\frac{1}{2\ddot{\Phi}}$, and $\ddot{\Phi}$ is the second-order dispersion. Substituting the Supplementary Equation 3 into the Supplementary Equation 4, we can obtain

$$E_{\mathrm{e}}(t)=G(2bt)\times\exp\left(jbt^{2}\right) \tag{5}$$

where $G(\omega)$ is the Fourier transform of $g(t)=E_{\mathrm{MRR\_DC}}(t)\times\exp\left(j(a+b)t^{2}\right)$. Then, the temporal waveform at the event plane can be obtained by the Supplementary Equation 5.

The key parameters in the simulations are listed as follows. The wavelength of the probe light is 1550 nm, the frequency interval and 3-dB bandwidth of the flat optical frequency comb are 10 GHz and 2 nm, respectively, the free spectral range (FSR) and 3-dB bandwidth of the ET-MRR are 8 nm and 0.15 nm, respectively, the slope a and the period of the sawtooth voltage are $40/\mathrm{ns}^2$ and 5 ns, respectively, the second-order dispersion of the normal dispersion is -1.92×10^{3} ps^2. As shown in Supplementary Fig. 2, the temporal gaps are obtained and the continuously cloaking window is up to 3.396 ns, which accords well with the experimental result.

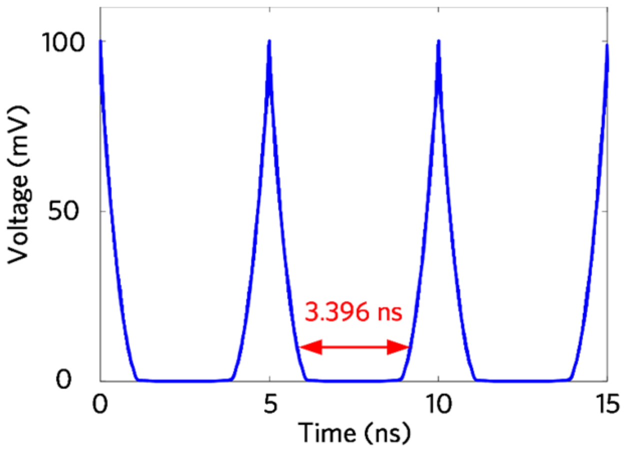

Supplementary Figure 2 | Corresponding simulated intensity distribution at the event plane.

Subsequently, we simulated the output temporal waveforms at four kinds of states of the temporal cloak. The event pulse is simulated with 33% dark return-to-zero (RZ) modulation format at 200 Mbit/s bit rate, and its eye diagram (green) is shown in Supplementary Fig. 3a. And the second-order dispersion of the dispersion compensation fiber is set as 1.92×10^{3} ps^{2}. The temporal output waveform of the temporal cloak system is recorded by a photodetector (PD) with 5 GHz bandwidth (See Supplementary Note 4). When the temporal cloak is turned off (namely, the driving signal of the ET-MRR is set to DC voltage and ensure that the resonance wavelength of the ET-MRR is aligned with the wavelength of the probe light) and the event is turned on, a clear eye diagram (blue) is obtained and is shown in Supplementary Fig. 3a. Due to the dispersion accumulation of dispersion compensation fiber, there is slight broadening of the blue eye diagram compared to the green eye diagram of the event. Then, we turn on the cloak and hold on the event. A CW (red) is recorded and shown in Supplementary Fig. 3a, showing the event is well hidden. As long as the event is turned off, whether cloak is turned on (black) or off (pink), the temporal output waveform is the same continuous waveform and those waveforms are shown in Supplementary Fig. 3b. All the above simulation results show that our solution is feasible to obtain a good temporal cloak.

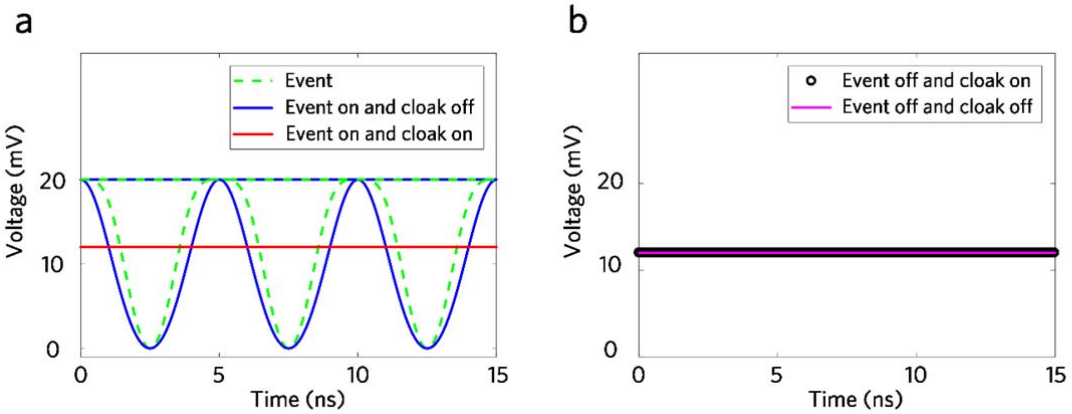

Supplementary Figure 3 | Simulation results of the temporal cloak. (a) Waveform of the emulated event and temporal out when event is on. (b) Temporal out when event is off.

Supplementary Note 4: Characteristic curve of the ET-MRR

To characterize the ET-MRR, different DC voltages are applied on it. When DC voltage with 0.8 V is applied on the MRR, the 3-dB bandwidth is 0.15 nm and the resonance wavelength is around 1548.297 nm, while the 3-dB bandwidth is enlarged to 0.46 nm and the resonance wavelength experiences blue shift of 1.572 nm when the applied DC voltage is increased to 1.3 V. The characteristic curve of the resonance wavelength versus the voltage is shown in Supplementary Fig. 4a. One can see that as the applied voltage increases, the 3-dB bandwidth increases and the resonance wavelength experiences blue shift. We also notice that when the driving voltage increases, the ET-MRR transmission at the peak would decrease, as shown in Supplementary Fig. 4b. When driving voltage is 0.8 V, the peak power is -27.8 dBm. The peak power is reduced to -37.6 dBm while the driving voltage is set to 1.3 V. The difference of the transmission peak is up to 10 dB, when the driving voltage varies from 0.8 V to 1.3 V.

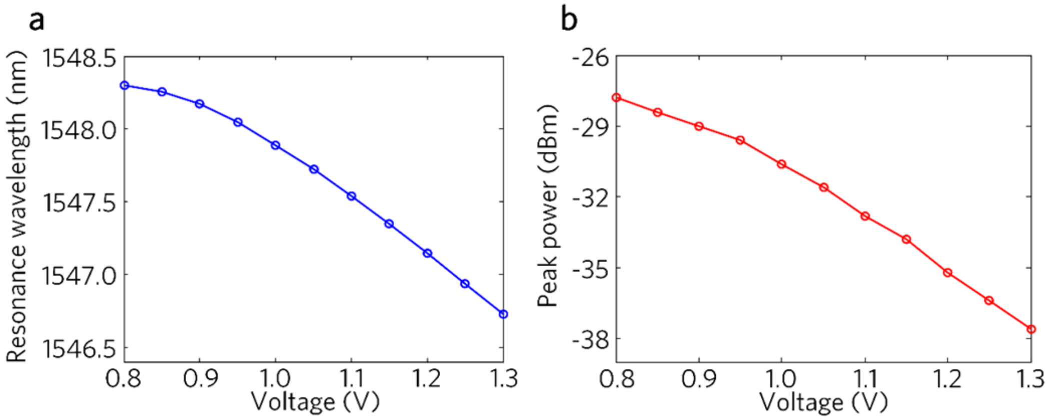

Supplementary Figure 4 | Characteristic curve of the ET-MRR. (a) Wavelength shift when the voltage is increased. (b) The curve of the resonance peak power versus the voltage.

Supplementary Note 5: Experimental setup

The experimental setup for temporal cloak system is shown in Supplementary Fig. 5. A CW light is emitted by a tunable laser source with a tuning resolution of 0.01 nm, which allows to align to the resonance wavelength of the ET-MRR precisely. Then the CW light is converted to a broadband optical frequency comb by the phase modulator (EOSPACE PM-5V4-40-PFA-PFA-UV, PM) with a strong radio frequency (RF) driving signal, and a wave shaper (Finisar 1000S) is used to tailor the output spectrum to a clival optical frequency comb. Subsequently, the clival optical frequency comb is coupled into the ET-MRR using vertical grating coupling method. The ET-MRR is driven by a sawtooth waveform generated by an electrical arbitrary waveform generator (Keysight M8195A, EAWG). A Bias-Tee is used to add a DC voltage to the sawtooth voltage so that the maximum and minimum voltages of the sawtooth waveform are 1.3 V and 0.8 V, respectively. Thus, the ET-MRR acts as a swept-frequency filter, just covering the spectral range of optical frequency comb. An 80 km single mode fiber with a total dispersion of 1340.5 ps/nm is used to open the cloaking window. Another 10.3 km dispersion compensating fiber with a total dispersion of -1342

ps/nm is used to close the cloaking window. An erbium doped fiber amplifier (EDFA) at the drop-port of the ET-MRR is used to compensate the fiber to chip loss. The output signal is received by a PD with 5 GHz bandwidth and recorded by digital communication analyzer (Agilent 86100C). It should be noted that the 3-dB bandwidth of ET-MRR is 57.5 GHz (when the drive voltage with 1.3 V), much larger than frequency interval of input optical frequency comb (10 GHz). Thus, beating signal with serious ripple may occur for the detected signal. This ripple can be removed by an electrical low-pass filter with a proper bandwidth, meanwhile the detection of the event is still undistorted within this proper bandwidth. Thus, the employed PD with 5 GHz bandwidth is very effective as a cloaking enhancer.

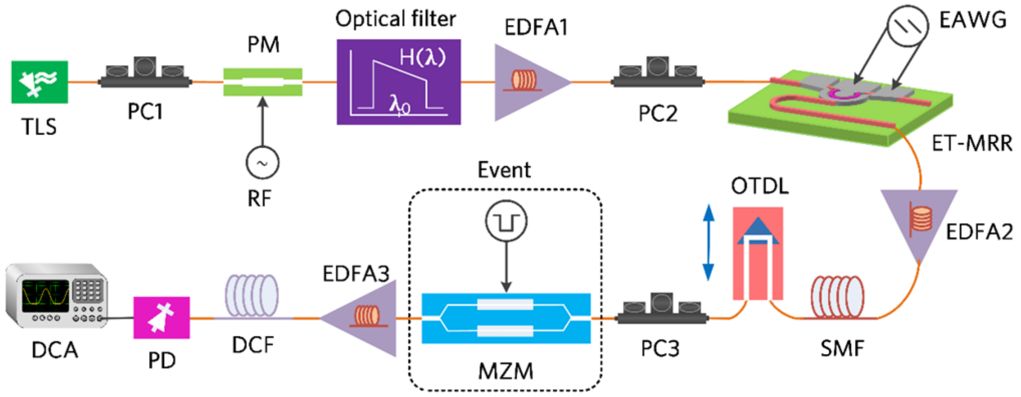

Supplementary Figure 5 | Experimental setup. TLS, tunable laser source. PC, polarization controller. PM, phase modulator. RF, radio frequency. EDFA, erbium doped fiber amplifier. EAWG, electrical arbitrary waveform generator. SMF, single mode fiber. OTDL, optical tunable delay line. MZM, Mach-Zehnder modulator. DCF, dispersion compensating fiber. PD, photodetector. DCA, digital communication analyzer.

The switch state of cloaking is controlled by applying an electrical split sawtooth signal on the ET-MRR and disabled by applying a DC electrical signal. The occurrence of event is emulated by a Mach-Zehnder modulator (JDSU 21067769-002, MZM). When the MZM is modulated by electrical dark RZ signals, the event is turned on. Noted that a spool of SMF is used to compress the waveform in time, and a temporal gap is opened
